# Protein-protein interactions in paralogues: Electrostatics modulates specificity on a conserved steric scaffold

**DOI:** 10.1371/journal.pone.0185928

**Published:** 2017-10-10

**Authors:** Stefan M. Ivanov, Andrew Cawley, Roland G. Huber, Peter J. Bond, Jim Warwicker

**Affiliations:** 1 Manchester Institute of Biotechnology, School of Chemistry, The University of Manchester, Manchester, United Kingdom; 2 Bioinformatics Institute, Agency for Science, Technology and Research (A*STAR), Matrix, Singapore, Singapore; 3 Department of Biological Sciences, National University of Singapore, Singapore, Singapore; Indian Institute of Science, INDIA

## Abstract

An improved knowledge of protein-protein interactions is essential for better understanding of metabolic and signaling networks, and cellular function. Progress tends to be based on structure determination and predictions using known structures, along with computational methods based on evolutionary information or detailed atomistic descriptions. We hypothesized that for the case of interactions across a common interface, between proteins from a pair of paralogue families or within a family of paralogues, a relatively simple interface description could distinguish between binding and non-binding pairs. Using binding data for several systems, and large-scale comparative modeling based on known template complex structures, it is found that charge-charge interactions (for groups bearing net charge) are generally a better discriminant than buried non-polar surface. This is particularly the case for paralogue families that are less divergent, with more reliable comparative modeling. We suggest that electrostatic interactions are major determinants of specificity in such systems, an observation that could be used to predict binding partners.

## Introduction

The interplay between biopolymers is critical in directing and maintaining physiological processes. Whilst genome-sequencing projects are providing large amounts of protein sequence data from many organisms, our understanding of binding specificity between proteins, and how a protein selects partners from closely related alternatives, remains limited. The majority of work in identifying specificity determinants focuses on the sequences and structures of the proteins involved. Methods for identifying residues that determine specificity face challenges, often due to an absence of suitable experimentally determined structures or the lack of affinity data [1]. Where structural models are available, computational predictions of protein—protein interactions focus on aspects of the association such as size, shape, and physicochemical complementarities at the interaction interface [2,3], as well as the factors that discriminate specific and non-specific interactions [4] Increasingly, experimental data are being combined with physicochemical calculations to provide predictions of interfaces and the roles of individual residues at interfaces [5,6] and, in turn, experiments are being guided by such calculations [7]. Sequence, evolutionary, and expression data may also be included in predictions [8]. Computational methods can be benchmarked against experimentally-determined complexes in community-wide studies [9,10].

Genomic and proteomic studies have shown that most proteins belong to families of evolutionarily, and often functionally, related molecules [11]. The number of proteins in a given family increases through gene duplication and the resulting generation of paralogues. For example, the human genome encodes several hundred protein kinases, which are believed to have arisen through large- and small-scale genetic duplications [12]. When interactions between proteins in paralogue families are considered, maintaining physiological cellular signaling requires proteins to distinguish between highly similar surfaces. Several approaches have been taken in attempting to rationalize such intricate interactions. Coexpressed proteins are enriched for interacting pairs [13], and within those pairs there may exist coevolving sequence signatures for the interaction [14]. Structural and bioinformatics studies have shown that protein–protein interfaces can be divided into a core and rim, with the rim being enriched in subfamily-specific residues [15]. There have been attempts to rationalize specificity through computational studies at differing levels of theoretical sophistication. Fong and Keating [16] have assessed the binding feasibility of different pairs of leucine zipper transcription factors by representing each pair as a multidimensional vector, the entries of which represent the different amino acid pairings from the two opposing chains. Each vector is then multiplied by a vector of corresponding weights for the different pairings. Most interfaces though are more complicated than the coiled-coil of a leucine zipper dimer, and are less amenable to such an approach. Atomistic models are, therefore, more prominent in rationalizations of specificity determinants. Calculations of electrostatic interactions with Generalized Born or Poisson-Boltzmann methods, combined with surface area, are often used in molecular mechanics, and have proven successful in identifying specificity determinants and recognition mechanisms [17,18]. Due to the extent of interfaces of even small protein–protein complexes, such methods are generally more successful in rationalizing protein–small molecule binding, than protein–protein binding [19]. More computationally expensive higher-level theory calculations, such as density functional theory and quantum mechanics, are almost exclusively carried out on protein–small molecule systems [20].

The present report examines specificity in paralogous protein–protein interactions from a structural viewpoint, combining atomistic-level detail, with rapid calculation of electrostatic interactions and surface burial. In computing interfacial properties, an empirical calculation approach is taken, using the solvent accessible surface area (SASA) approach of Lee and Richards [21] and a Debye-Hückel computation of charge interactions between groups bearing net charge [22]. Computed properties are compared between interacting and non-interacting pairs of proteins, identified from literature. This study aims to establish whether these simple interface descriptors discriminate between binding and non-binding pairs in paralogous protein–protein interactions. Sets of experimental data have been identified, together with structural templates for modeling paralogue complexes, so that this hypothesis can be tested. Perhaps the most clear-cut example is in transcription factor heterodimerization *via* leucine zippers, where charge interactions modulate specificity on a relatively conserved steric framework [23]. The simple surface area and electrostatics model allows rapid estimation of interfacial energetics over a wide range of paralogue complexes generated by sidechain replacement comparative modeling. It is found that the leucine zipper model for charge mediated specificity persists in other systems, although both the effect and the confidence with which it can be assessed falls away the further that sequences diverge between template and modeled proteins. Whilst there are many examples of paralogue family protein—protein interactions, corresponding experimental data are limited. Improved modeling of specificity in such interactions will lead to a better understanding of structure—function relationships, and protein—protein interaction networks.

## Methods

### Sequence alignment and comparative modeling

The key requirements for a system to be included in this study are the availability of binding data, and the presence of at least one representative complex in the protein structural database [24]. After obtaining a three-dimensional structure of a complex, a multiple sequence alignment is generated between each molecule in the template and the relevant set of paralogues. Sequences were obtained from UniProt [25]. Sequence alignment was performed with the default settings of T-Coffee [26], and used in generating a three-dimensional structure for each possible combination of potential interactors. The comparative modeling pipeline incorporated side-chain replacement with fixed backbones. Identical side-chains between template and model are maintained in their conformers, while swapped side-chains are repacked [27] with an adaptation [28] of a self-consistent mean-field method for rotamer selection from a rotamer library [29]. The algorithm performs pairwise packing of rotamers while observing a predefined tolerance for clashes of van der Waals radii. Beyond that tolerance, overlap of atomic van der Waals radii is prohibited subject to a further relaxation that is incremented until a packing solution is found i.e. with all sidechains having at least one allowed rotamer [28].

### Buried surface and electrostatic energy calculations

The estimated electrostatic energy of interaction for groups bearing net charge (NetQ) and changes in non-polar and polar solvent accessible surface areas (ΔSASAnp and ΔSASApol) are calculated for all complexes modeled as rigid structures, with the differences for surfaces denoting subtraction of the sum of the component values from the complex value. Each component may be one, or more than one, polypeptide chain [27]. Surfaces are calculated using a sphere of radius 1.4 Å rolling on the van der Waals contour of a protein [21,28]. In keeping with the empirical nature of this study, a framework for electrostatic interactions was used that allowed rapid application to multiple comparative models, with simple Debye-Hückel estimation of charge interactions in water at neutral pH and 0.15 M ionic strength [22]. For each complex, NetQ is computed by summing all interactions between charged groups (Lys, Arg, N-terminus +1; Asp, Glu, C-terminus -1). This is achieved by calculating charge interactions in the complex and subtracting charge interactions in the separated components, thus giving the resultant charge contribution to complexation. Charges q_i_ and q_j_, separated by a distance of r, interact with a 1/r Coulomb potential in a dielectric medium with the relative permittivity of water (80), modified by a Debye-Hückel factor at 0.15 M ionic strength [22].

### Binding data and structural templates

Experimental data obtained from literature are used to separate interactors from non-interactors, which are then coupled with template-based comparative models for the potential interacting pairs. In the case of bZIPs, a dataset of 127 strong interactions, 324 weak interactions, and 1214 non-interactions was assembled from a comprehensive study of leucine zipper dimerization [30]. The authors defined the interactions as: strong with a z-score (number of standard deviations from the mean) for signal > 10, weak with a z-score between 2.5 and 10, and non-interactors—a lower z-score. Leucine zipper sequences were aligned with each other and the template from the first zipper anchoring position. Templates with long helical regions were chosen, 1T2K [31] and 1CI6 [32].

The *Caulobacter crescentus* genome encodes three parE toxins and one pseudogene (parE_2_) and their corresponding parD antitoxins [33], whereas the relEB family is represented by four toxin-antitoxin pairs [34]. The parED/relEB superfamily toxins and antitoxins interact with each other on a 1:1 basis [35], i.e. each toxin interacts with and is neutralized by its cognate antitoxin only. Thus, there are 3 interacting and 6 non-interacting pairs in the parED system, and 4 interacting and 12 non-interacting pairs in the relEB system. Another toxin–antitoxin system is the *Mycobacterium tuberculosis* vapBC family, comprising 48 vapC toxins that interact on a 1:1 basis with their vapB antitoxins [36], which produces 48 interacting and 2256 non-interacting pairs. Complex structures for the toxin—antitoxin pairs were generated by modeling on 3KXE [35] for the parED family; 2KC8 [37] for the relEB family; and 3H87 [38] and 3DBO [39] for the vapBC family.

As part of the ubiquitination pathway, ubiquitin-conjugating enzymes (E2s) interact with ubiquitin-ligating enzymes (E3s). Human E3 ubiquitin ligases are divided into three subgroups depending on the structure of the catalytic domain, the largest group being the RING-type E3s [40]. In a genomic study, 31 human E2s, 17 E2 pseudogenes, and 313 RING-type E3s were identified [41]. A dataset of 329 interactions and 7219 non-interactions was derived. Two template structures of different RING domain lengths were used: 3HCT, 40 amino acids [42] and 4CCG, 59 [43]. A separate study on functional interactions between 22 human E2s and 9 HECT type E3s produced a dataset of 94 interacting and 104 non-interacting pairs [44]. We generated all 198 models using the 3JVZ [45] and 5HPT [46] template structures.

Interaction data on BH3 peptide interactions with antiapoptotic proteins, consisting of 48 IC50 values, was obtained from solution competition assays on the binding between five antiapoptotic proteins and BH3 peptides from 10 proapoptotic proteins [47]. The 48 complexes were generated with comparative modeling based on the 2XA0 [48] template. Table 1 provides a summary of the systems examined in this work.

**Table 1:** Summary of systems studied.

system		interactors (N1)	weak interactors	non-interactors (N2)	experimental technique [reference]
bZIPs		127	324	1214	fluorescent peptide arrays [30]
E2 –RING E3s		329	-	7219	yeast two-hybrid screen (Y2H) [41]
E2 –HECT E3s		94	-	104	functional screen [44]
Toxins–antitoxins	parE–parD	3	-	6	growth inhibition [35]
	relE–relB	4	-	12	growth inhibition [35]
	vapC–vapB	48	-	2256	growth inhibition and Y2H [36]
Bcl-2-intrafamily interactions		43	-	5	solution competition assay [47]

Before proceeding to comparative modeling, we examined the binding mode between different pairs of proteins within each system for conservation. Structural and other experimental data demonstrate that the binding modes within the Bcl-2 family and the E2—E3 system are highly conserved [49,50]. Our models of Bcl-2 family complexes are in excellent agreement with recently published structures [51] with Cα RMSDs ~ 0.5 Å. Only in the toxin-antitoxin systems did we observe large divergence in sequence and structure, with sequence identities as low as 4% and RMSDs above 3 Å.

Where comparisons are made between sets of calculated properties, statistical significance is assessed with the two-tailed Mann-Whitney U test, a non-parametric test used to determine whether samples derive from populations with the same distribution. Use of multiple templates allowed us to assess the robustness of our results.

## Results

### Workflow

**Fig 1:**
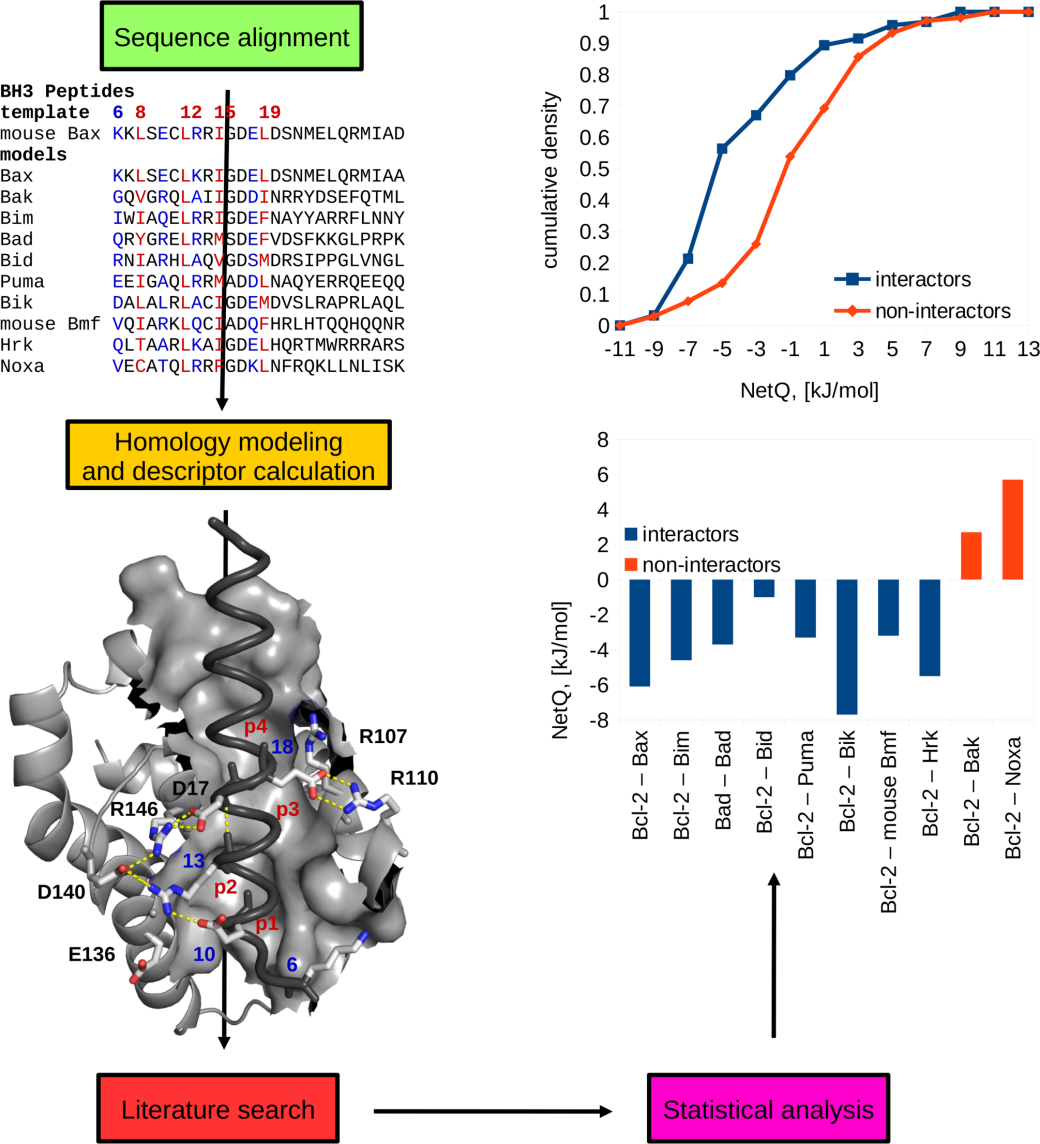
Schematic representation of the workflow. In this example of BH3 peptides potentially binding to the Bcl-2 antiapoptopic protein, multiple sequence alignment feeds into comparative modeling, generation of electrostatic and buried surface area interface descriptors, and subsequent comparison between interactors and non-interactors, as individual complex and cumulative density data. The cumulative density derives from a larger dataset than the sequences shown. Key hydrophobic residues in the sequence alignment are highly conserved and highlighted in red. These four positions fit into conserved hydrophobic pockets on the surface of the protein, labeled (in red), p1, p2, p3, and p4. A surface representation is shown for the groove, superimposed on backbone representations for other parts of the complex, dark grey for Bax and light grey for Bcl-2. Variable positions (6, 10, 13 and 18) discussed in the text are indicated in blue in the sequence alignment and with stick representations in the structure. Also shown in stick representation and labeled are the invariant aspartic acid in position 17 and key residues from the Bcl-2 protein. Interside chain salt bridges and backbone hydrogen bonds in the template structure are represented with dashed lines.

A multiple sequence alignment between paralogues in protein families is used to perform comparative modeling with one or more template structures for a complex. Fig 1 shows the procedure for 10 BH3 peptides and a template structure of an antiapoptotic protein bound to a BH3 peptide. For each of the 10 modeled complexes, interface descriptors are computed: interactions of groups bearing net charge (NetQ), change in non-polar solvent accessible surface area upon complex formation (ΔSASAnp), and change in polar solvent accessible surface area (ΔSASApol). Interacting and non-interacting pairs are identified from literature and interfacial properties are compared between the two groups, with appropriate statistical analysis. Results are plotted, for this example (Fig 1) as individual values of NetQ for interacting and non-interacting pairs in the Bcl-2 –BH3 peptide set, and also as the cumulative density of NetQ values in a larger dataset.

### Basic leucine zipper transcription factors

**Fig 2:**
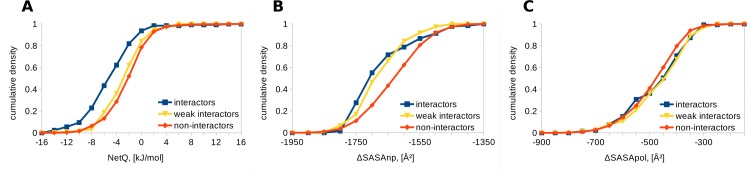
Comparison of interfaces for bZIP leucine zippers. Cumulative densities for interactors, non-interactors and 324 weak interactors [30] are shown, using the 1CI6 template. A. NetQ. B. ΔSASAnp. C. ΔSASApol.

A challenge for studies that seek to understand interaction specificities between paralogue families of proteins is the availability of high quality experimental data. Such data are available for the basic leucine zipper transcription factors (bZIPs), upon which Newman and Keating have carried out a comprehensive binding study [30]. After performing a multiple sequence alignment, 3-dimensional models of all possible binary combinations of bZIPs were generated. Interfacial properties for the different complexes were calculated and compared between interactors and non-interactors. The electrostatic energy of interaction (NetQ) is more favorable for interactors, (mean M = -5.3, standard deviation SD = 3.9 kJ/mol, number of interacting pairs = N1 = 127), than for non-interactors (M = -2.3, SD = 3.40 kJ/mol, number of non-interacting pairs = N2 = 1214), when modeling on the 1CI6 template. Change in non-polar solvent accessible surface area is larger in interactors (M = -1681, SD = 99 Å^2^) than non-interactors (M = -1633, SD = 95 Å^2^), whereas change in buried polar accessible surface area is similar for interactors (M = -473, SD = 112 Å^2^) and non-interactors (M = -486, SD = 100 Å^2^) (Fig 2). The NetQ and ΔSASAnp differences between interactors and non-interactors are significant, with p values of 4.29x10^-19^ and 1.27x10^-9^ respectively using the two-tailed Mann-Whitney U test, whereas ΔSASApol is not significantly different (p = 0.88). Weak interactors are located between interactors and non-interactors, although closer to interactors for ΔSASAnp and closer to non-interactors for NetQ. The ranking of p-values is the same when modeling with the 1T2K template, with Mann-Whitney test p-values for interactors compared with non-interactors of 6.99x10^-14^ for NetQ, 2.71x10^-10^ for ΔSASAnp and 2.62x10^-5^ for ΔSASApol.

### E2 ubiquitin conjugating enzymes–RING E3 ubiquitin ligases

Ubiquitination contributes to the regulation of many physiological processes [52]. The transfer of ubiquitin to a protein substrate in the cell occurs through a complex series of interactions involving E1, E2 and E3 enzyme classes, with the number of enzymes in each class increasing along the pathway. E2 enzymes accept activated ubiquitin from E1s and are, in turn, recognized by an E3 ubiquitin ligase. Finally, E3s transfer the ubiquitin to a protein target [53]. Experimental studies on the ubiquitination pathway have provided insight into the specificity of protein-protein interactions within the system [41].

The majority of suitable templates in the Protein Data Bank (PDB) [24] represent 36–46 residue-long RING domains. Modeling on a template with a RING domain length of 40 amino acids (3HCT) gave all three properties, NetQ, ΔSASAnp, and ΔSASApol, as significantly different between interactors and non-interactors. NetQ for interactors of M = -2.1, SD = 2.3 kJ/mol compares with M = 0.6, SD = 3.0 kJ/mol for non-interactors (N1 = 329, N2 = 7219, Mann-Whitney p = 4.70x10^-22^). For interactors, ΔSASAnp, M = -661, SD = 77 Å^2^ compares with M = -610, SD = 102 Å^2^ for non-interactors (p = 1.31x10^-22^). For ΔSASApol, interactors give M = -370, SD = 98 Å^2^ and non-interactors M = -398, SD = 95 Å^2^ (p = 5.76x10^-9^).

**Fig 3:**
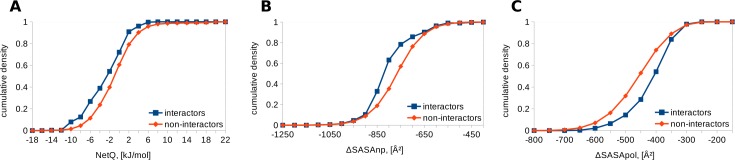
Comparison of interfaces for E2 –RING E3 complexes modeled on 4CCG. Cumulative densities for interactors and non-interactors are shown. A. NetQ. B. ΔSASAnp. C. ΔSASApol.

The largest available E3 structure suitable to be a template, a 59 residue-long RING domain bound to an E2 enzyme (4CCG) also gave separation of all three properties (Fig 3). NetQ for interactors is M = -3.0, SD = 4.1 kJ/mol and for non-interactors, M = -0.9, SD = 4.4 kJ/mol, with Mann-Whitney p = 1.35x10^-13^. For ΔSASAnp, M = -806, SD = 99 Å^2^ for interactors compares with M = -767, SD = 100 Å^2^ for non-interactors (p = 9.50x10^-18^). For ΔSASApol, interactors give M = -419, SD = 74 Å^2^ and non-interactors M = -460, SD = 90 Å^2^, with p = 1.65x10^-18^.

### E2 ubiquitin conjugating enzymes–HECT E3 ubiquitin ligases

**Fig 4:**
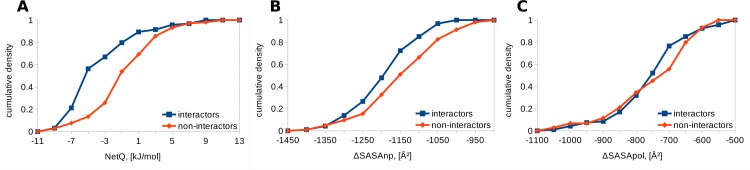
Comparison of interfaces for E2 –HECT E3 complexes modeled on 5HPT. Cumulative densities for interactors and non-interactors are shown. A. NetQ. B. ΔSASAnp. C. ΔSASApol.

**Fig 5:**
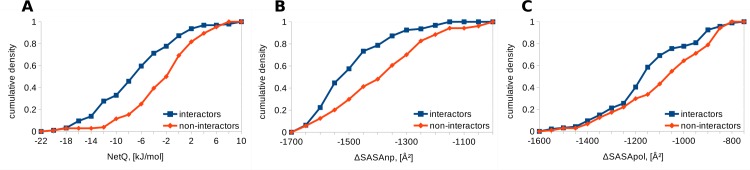
Comparison of interfaces for E2 –HECT E3 complexes modeled on 3JVZ. Cumulative densities for interactors and non-interactors are shown. A. NetQ. B. ΔSASAnp. C. ΔSASApol.

HECT E3 ubiquitin ligases, like the RING E3s, are involved in transferring ubiquitin from an E2 enzyme to a protein target. A study on functional E2 –HECT E3 interactions provides interaction data [44]. Using the 5HPT template (Fig 4), NetQ is more favourable for interactors (M = -4.0, SD = 4.1 kJ/mol) than for non-interactors (M = -1.0, SD = 4.0 kJ/mol), which is statistically significant with the two-tailed Mann-Whitney U test (N1 = 94, N2 = 104, p = 6.39x10^-8^). Buried non-polar surface is significantly larger in interactors (M = -1198, SD = 86 Å^2^) than non-interactors (M = -1153, SD = 105 Å^2^, p = 2x10^-3^), whereas polar surface is not significantly different (interactors M = -758, SD = 113 Å^2^, non-interactors M = -751, SD = 123 Å^2^, p = 0.33). Similar results are obtained with the 3JVZ template (Fig 5) listing interactors *versus* non-interactors: NetQ, M = -7.1, SD = 6.3 kJ/mole *versus* M = -2.9, SD = 5.6 kJ/mol, with p = 2.87x10^-7^; ΔSASAnp, M = -1501, SD = 123 Å^2^ versus M = -1397, SD = 165 Å^2^, with p = 4.35x10^-6^; ΔSASApol, M = -1159, SD = 177 Å^2^ versus M = -1091, SD = 190 Å^2^, with p = 5.39x10^-3^. For the 3JVZ template, unlike 5HPT, buried polar surface area is also significantly different, possibly because the C-lobe of the HECT domain is positioned differently, capturing different points along the pathway of transferring ubiquitin from the E2 to the E3.

### Toxin–antitoxin pairs

**Fig 6:**
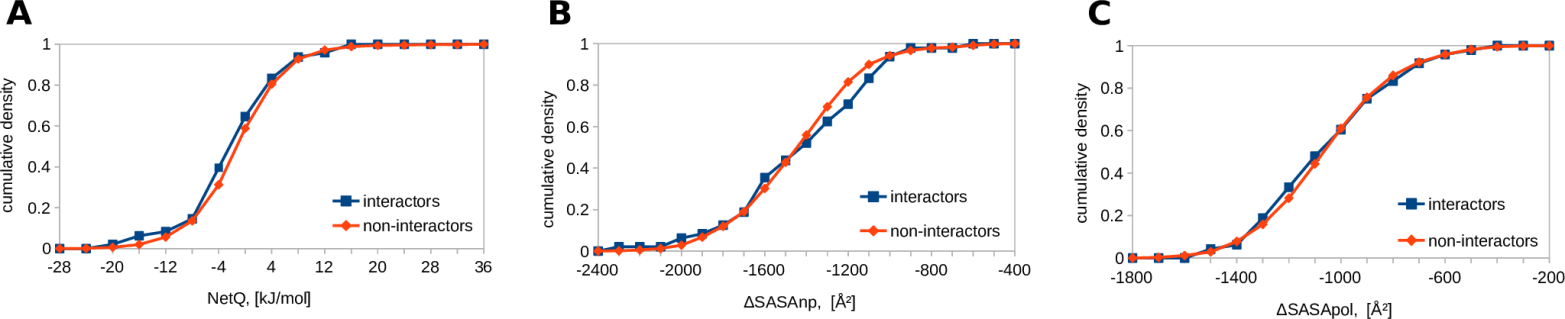
Comparison of interfaces for vapC toxin–vapB antitoxin complexes modeled on 3H87. Cumulative densities for interactors and non-interactors are shown. A. NetQ. B. ΔSASAnp. C. ΔSASApol.

Specificity data are available for parD-parE pairs in *Caulobacter crescentu*s [35], and vapB-vapC pairs for the related vapBC system in *Mycobacterium tuberculosi*s [36]. NetQ, ΔSASApol and ΔSASAnp are not significantly different between interactors and non-interactors for the vapBC family (N1 = 48, N2 = 2256, Fig 6) when modeling on the 3H87 or 3DBO templates. Modeling parE–parD pairs (N1 = 3, N2 = 6) on the 3KXE template, and relE–relB (N1 = 4, N2 = 12) on the 2KC8 template, also fails to produce any separation between interactors and non-interactors. Toxin–antitoxin pairs are by far the most divergent system, with sequence identities as low as 4% within the toxin or antitoxin families, and Cα RMSDs between 3 and 7 Å for aligned template structures.

### Bcl-2-family proteins

**Fig 7:**
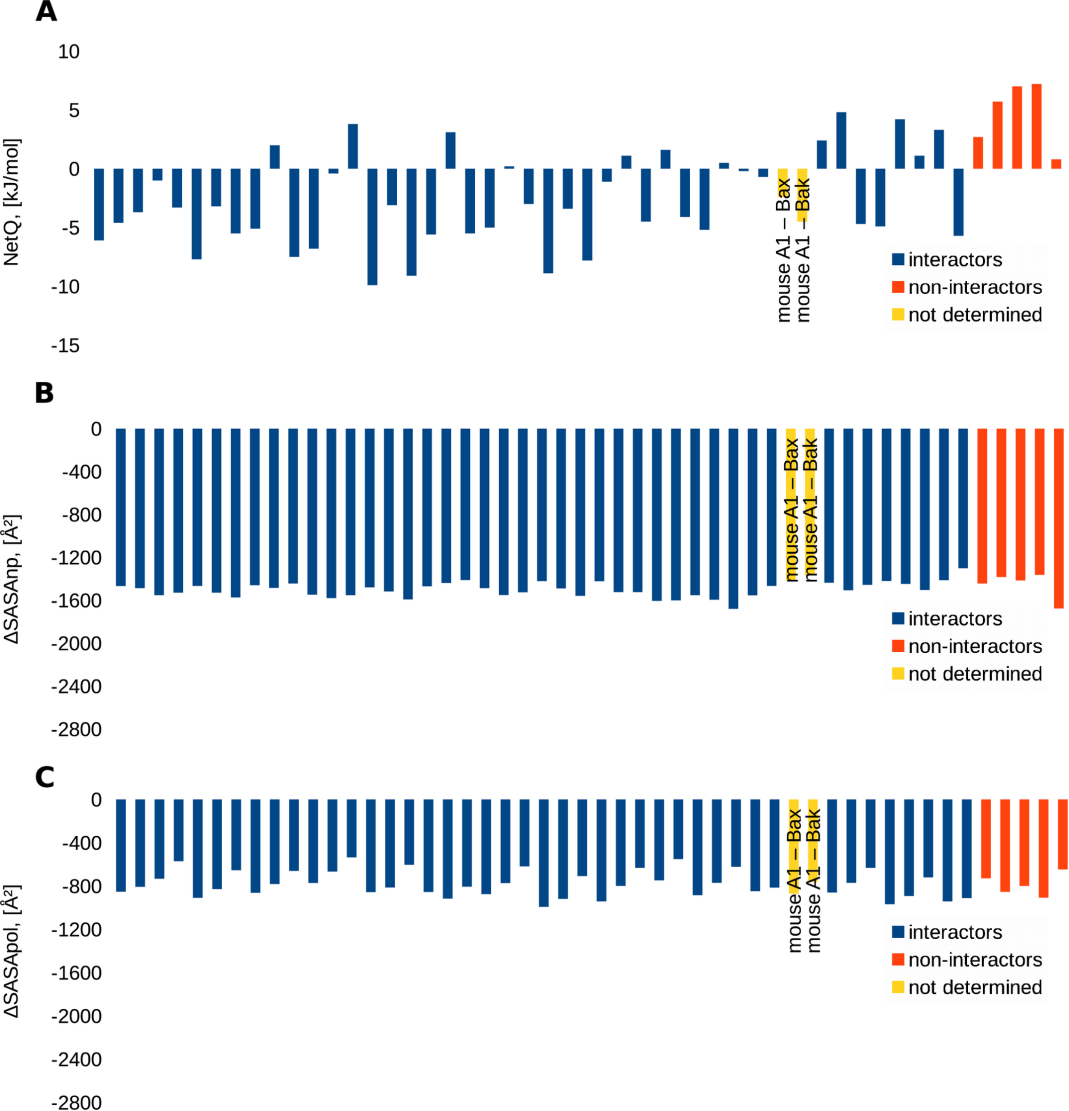
Comparison of interfaces for BH3 peptide–binding groove interactions, modeled on 2XA0. Color-coded histograms for interactors (blue), non-interactors (red), and interactions that have not been determined (yellow). A. NetQ. B. ΔSASAnp. C. ΔSASApol.

Interactions among the Bcl-2-like proteins are crucial in regulating apoptosis. Specificity data are available for a set of BH3 peptides interacting with BH3-binding grooves [47]. After modeling antiapoptotic protein–BH3 peptide interactions on a template of human Bcl-2 bound to a BH3 peptide (2XA0), and comparing charge interactions and buried surfaces between interacting (N1 = 43) and non-interacting pairs (N2 = 5), the most evident difference is that non-interactors typically have a less favorable NetQ than interactors (p = 0.002, Fig 7). Buried surface is less discriminating between interactors and non-interactors (p = 0.131 for ΔSASAnp, p = 1 for ΔSASApol).

## Discussion

This study assesses to what extent interactions between groups bearing net charge correlate with specificity for complexes formed by families of paralogous proteins at a common interface. Modeling paralogues on a suitable template and comparing empirical interface properties produces significant separation between interactors and non-interactors in most systems, with electrostatic interactions (between groups bearing net charge) being most discriminatory, followed by buried non-polar surface, with buried polar surface being least discriminatory. It is shown that the results are largely independent of the template, although there is a limit to the template-based modeling with our current methods, demonstrated by the bacterial toxin–antitoxin pairs. These systems have diverged sufficiently to seriously impact on the accuracy of the comparative modeling process. For example, the vapB_2_–vapC_2_ and vapB_5_–vapC_5_ pairs have an overall sequence identity of 6%, and an RMSD between template structures of 6.6 Å, in contrast to a more typical case in the current work of sequence identities ~ 45% and RMSDs ~ 1.5 Å. Extensive sequence divergence, seen particularly in bacterial systems, is likely to provide a challenge for even the most sophisticated comparative modeling tools [54]. However, the lower sequence divergence seen for proteins in paralogue families in metazoan systems make them amenable to the comparative studies that we have employed.

Our sidechain replacement comparative modeling tool provides no opportunity to model insertions and deletions. Whether such changes can be modeled with sufficient accuracy and speed for large-scale analysis of complexes remains an open question. An available option is whether to repack all sidechains or to employ a more minimal repacking of only those sidechains that differ between model and template. The minimal repacking scheme has been used, since amino acid conservation could reflect an important role in maintenance of structure [55]. For example, with BH3 peptides, 4 conserved hydropobic residues bind into 4 conserved pockets on the antiapoptotic proteins, and an invariant aspartic acid forms a salt-bridge with a conserved arginine from the partner protein (Fig 1). In RING E3s, conserved histidine and/or cysteine residues coordinate Zn^2+^ to maintain the native protein structure. It has been found that preserving the template amino acid sidechain rotamer is beneficial in maintaining the stability of modeled antiapoptotic protein–BH3 peptide complexes during molecular dynamics simulations [18].

High throughput experimental data for protein–protein interactions are key for the current study, but these data can be imprecise. For example, the largest dataset used, E2 –RING E3 interactions, derives from a yeast two-hybrid screen [41]. Given the general low affinity of E2 –E3 interactions [56], the screen may contain false positive and/or false negative data. Additionally, the functional assay used in the E2 –HECT E3 study is not capable of detecting interactions which only extend ubiquitin chains on mono-ubiquitinated targets or require cofactors [44]. Further computational study would benefit from more data collection in a variety of paralogue systems.

In agreement with previous work [57], we find that non-polar surface constitutes the majority of the interface, consistent with it being the dominant contributor to the free energy of binding. The current study suggests that superposed on burial of non-polar surface, the interactions of groups bearing net charge are a major determinant of binding specificity, for interactions between members of paralogue families. This finding is consistent with the core and rim model of protein interfaces [58], which postulates that conservation is greatest at the mostly hydrophobic core [59][60]. Our study indicates that for specificity of protein interactions from paralogue families, at a common interface, charge alterations make a substantial contribution, on a relatively conserved steric scaffold.

This observation can be interpreted from the standpoint of the core and rim model, and co-evolution of sequences, at least in the case of BH3 peptide–antiapoptotic protein complexes. Anchoring hydrophobic resides are highly conserved in this system (Fig 1), with conservation of the amino acids forming the 4 non-polar pockets, and illustrated by the low variation of ΔSASAnp values (Fig 7B). Key variable residues are blue in the sequence alignment of Fig 1, and contribute to a charge-mediated specificity, evident in Fig 7A. These amino acids vary within the alignment between acidic, basic, and uncharged. It follows that co-evolutionary methods could be fruitfully employed in identifying interacting pairs through grouping into subsystems. It is possible to cast our current results in the context of the core and rim model. Here, the core is dominated by conserved non-polar amino acids, whilst more polar groups at the rim play a large role in determining specificity. It is apparent that significant variation of non-polar surface also occurs within most of the systems studied here, although in general changes in charge interactions are better at distinguishing interactors from non-interactors. For one system, BH3 peptide complexes with antiapoptotic proteins, we have discussed how co-evolutionary approaches could be applied to specificity determinants. Co-evolutionary methods are likely to be more generally applicable to these systems, and await further analysis. Using the BH3 peptide complexes, we illustrate one example of how variation in rim residues may currently be an under-recognised feature in specificity determination, as compared with the well-recognised hydrophobic pockets and conserved aspartic acid. Mutation of E18 in Bim to a serine diminishes the binding of Bcl-xL, whereas phosphorylating the resultant serine restores binding, as a result of phopshoryl group interactions with arginine residues [61]. We have previously uncovered such behavior with molecular dynamics simulation and free energy calculations [18], but it is apparent in the current work that these patterns can also be recognized through more simple and much faster calculations.

We have established that charge-charge interactions contribute substantially to a fine-tuning of pair interaction specificity in the systems studied, and in one case show that this modulation is based largely in the rim of the core and rim model for protein-protein interactions. It is unclear why interactions evolve in this manner, although two lines of enquiry are apparent and could be further investigated. First, the effects of mutations (non-polar *versus* polar/charged residues) on stability of each interacting partner could lead to a preference for less deleterious charge swaps over non-polar surface alteration. Second, in a view of the crowded macromolcular environment, complementarity of charge interactions could afford a mechanism for scanning of potential partners at moderately longer range than the solvent exclusion of non-polar interactions.

The empirical modeling pipeline could be trialed with a combination of charge and surface burial, or inclusion of volume-based descriptors [27], and with other features, such as hydrogen bonding, more detailed analysis of buried surface and solvation [62], and alternate analysis of side-chain conformers in protein-protein interactions [63].Further work is required to establish the degree to which our empirical model can be used predictively for interacting and non-interacting pairs, in particular looking at restrictions imposed by divergence at the sequence alignment and comparative modeling stages. In this regard, we have included calculations for A1 –Bax and A1 –Bak binding, which were not present in the original experimental binding dataset. Our calculations suggest that these are favourable interactions, which is corroborated by experimental work for A1 –Bax [64] and A1 –Bak [65]. The benefit of the current study is that a very simple model is employed, so that the effectiveness of charge interactions in contributing to interaction specificity is clearly encoded in the geometry of charge disposition at the interface. Our study is designed around variation at a common interface, which yields to the simple model applied, in contrast for example to more detailed modeling for design of a new interface [66]. It be could applied to modeling those parts of protein—protein interaction networks within a cell [67,68,69] that involve interactions between proteins from paralogue families.
